# A Comparison of the Effectiveness of Nintedanib and Pirfenidone in Treating Idiopathic Pulmonary Fibrosis: A Systematic Review

**DOI:** 10.7759/cureus.54268

**Published:** 2024-02-15

**Authors:** Ruzhual K Man, Amaresh Gogikar, Ankita Nanda, Lakshmi Sai Niharika Janga, Hembashima G Sambe, Mohamed Yasir, Shivana Ramphall

**Affiliations:** 1 Research, California Institute of Behavioral Neurosciences and Psychology, Fairfield, USA

**Keywords:** dysregulated immune response, lung repair mechanism, dyspnoea, dlco, pirfenidone, nintedanib, forced vital capacity, idiopathic pulmonary fibrosis, interstitial lung disease

## Abstract

Idiopathic pulmonary fibrosis (IPF), which shares a radiographic pattern with the usual interstitial pneumonia (UIP), is a specific form of chronic and progressive interstitial lung disorder resulting in persistent fibrosis and impaired lung function. Most of the patients suffer from dyspnea which adversely affects health-related quality of life (HRQOL). The underlying etiology of the disease is not yet understood, but research done on the subject reveals that aberrant repair mechanisms and dysregulated immune responses may be the cause. It can affect any age group but predominantly affects patients who are above 50 years of age. It has been observed that in addition to age, the reasons are also related to smoking, pollution, and inhalation of harmful elements. As the cause of IPF is still unknown and there is no cure yet, presently, it is treated to delay lung function loss with antifibrotic medications, nintedanib, and pirfenidone. However, both nintedanib and perfenidone have side effects which affect different patients in different ways and with different levels of severity, thereby making the treatment even more challenging for medical practitioners. The present systematic review aims at studying the efficacy of pirfenidone and nintedanib in relieving symptoms and in extending survival in patients. A detailed search was done in relevant articles listed in PubMed, ScienceDirect, and the New England Journal of Medicine between 2018 and 2023. It was observed that the most accepted way of measuring the progression of IPF is the evaluation of pulmonary function by assessing the forced vital capacity (FVC). Several studies have shown that the decline in FVC over a period of 6-12 months is directly associated with a higher mortality rate. The outcomes were similar in both male and female irrespective of age, gender, and ethnicity. However, some patients being treated with pirfenidone and nintedanib experienced various side-effects which were mainly gastrointestinal like diarrhea, dyspepsia, and vomiting. In the case of pirfenidone, some patients also experienced photosensitivity and skin rashes. In cases where the side-effects are extremely severe and are more threatening than the disease itself, the treatment has to be discontinued. The survival rate in patients with IPF is marked by a median of 3-5 years that is even lower than many cancers; hence, the treatment should be started as soon as the disease is detected. However, further research is needed to establish the etiology of IPF and to establish treatments that can stop its progression.

## Introduction and background

Out of all the lung diseases known so far across all age groups, idiopathic pulmonary fibrosis (IPF) is challenging medical professionals and is a reason for fear for the patients as the lung tissues get permanently scarred in this condition, the breathing becomes increasingly difficult, and the survival rate is just between three and five years, and we still do not know the cause. Additionally, this disorder has been a chronic and progressive disorder of the interstitial lung [1,2]. 

Although IPF is detected more in patients above 50 years of age, the challenge lies in the fact that there is no minimum age for the diagnosis of this disease. It is also seen in younger populations as IPF development results due to complex interactions between genetic predisposition, environment, and dysregulated wound repair mechanisms within the lung tissues [2]. In older patients, the reasons are more likely to be a cumulative effect of the aging process, cigarette smoking, occupational hazards involving breathing in harmful elements, and environmental pollution [2,3]. As the median age of diagnosis of IPF is between 65 and 70 years, it is crucial to be able to grasp how the IPF impacts the elderly for better treatment [4].

The scientific explanation of the etiology of the disease is still unknown, but the research done on the subject so far reveals that fibrotic scar tissue is produced due to the stimulation of fibroblasts and myofibroblasts, which in turn produces an excessive amount of extracellular matrix protein [5]. According to the research on the subject, this happens due to an aberrant repair process kickstarted by repetitive microinjuries to the alveolar epithelium, pro-fibrotic signaling pathways, and dysregulated immune responses, resulting in persistent fibrosis and damaged respiratory health due to impaired lung function [6]. 

As the cause of IPF is still unknown and there are currently no cures, it is being treated using a multidisciplinary approach of relieving symptoms, slowing the progression of the condition, and improving the quality of life. The priority is to delay lung function loss, for which antifibrotic medications, like pirfenidone and nintedanib, have shown promising results in extending survival [7,8]. However, medical professionals are facing huge challenges in the use of these drugs for the treatment of IPF patients because, firstly, the mechanisms of these medications are still being investigated. Secondly, their effectiveness, tolerability, and safety vary from patient to patient and depend on how quickly the treatment is initiated [9,10]. 

In severe cases, lung transplantation, oxygen therapy, and pulmonary rehabilitation are also options that are considered, but what is urgently needed is targeted medicines that can not only halt the fibrotic process in IPF but reverse it. This comprehensive assessment of clinical research, randomized controlled trials, metanalyses, and review articles compares the efficiency of two therapeutic modalities in delaying the development of lung fibrosis and each of its accompanying functions. The goal is to expand the current small body of knowledge regarding IPF and its treatment by demonstrating which of these two drugs, nintedanib and pirfenidone, can clinically improve patient outcomes.

## Review

Methodology

**Figure 1:**
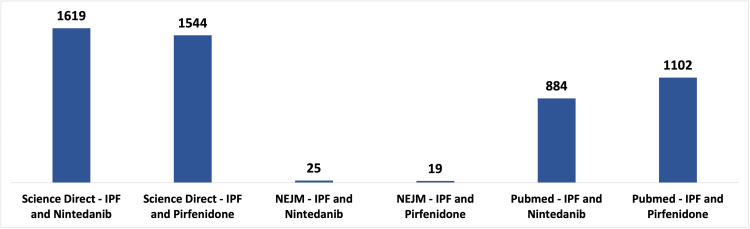
Keyword frequency across all databases collected between 2018 and 2023 ScienceDirect is the world's leading source for scientific, technical, and medical research; idiopathic pulmonary fibrosis (IPF); the New England Journal of Medicine (NEJM); PubMed comprises more than 36 million citations for biomedical literature, life science journals, and online books.

We conducted a comprehensive literature search in line with the Preferred Reporting Items for Systematic Reviews and Meta-Analyses (PRISMA) 2020 guidelines [11]. Utilizing a combination of Medical Subject Headings (MeSH) phrases and keywords such as "Idiopathic Pulmonary Fibrosis," "Nintedanib," "Pirfenidone," "IPF," and "Anti-fibrotic," we employed a Boolean approach to search for relevant publications on the effectiveness of antifibrotic therapy in IPF. The databases consulted included PubMed/MEDLINE, ScienceDirect, and the New England Journal of Medicine, exploring free and paid full-text articles listed between 2018 and 2023. The population of our study comprised patients diagnosed with IPF from various age groups, genders, and ethnicities who were undergoing treatment with nintedanib and pirfenidone. The outcomes of our extensive search across the three primary data sources are illustrated in Figure 1.

Criteria for Inclusion/Exclusion

Various criteria were taken into consideration for the study. The review focused on randomized control trials (RCTs) done between 2018 and 2023. The focus was majorly on patients above the age of 45 years. We included studies of patients of both sexes who were being treated with either nintedanib or pirfenidone or both drugs together. All studies focused only on RCTs done on humans. We excluded studies falling within the domain of grey literature and unpublished literature. We also excluded studies done on animals. Care was focused on checking for duplicates and their exclusion from the study. Table 1 depicts the criteria for inclusion and exclusion in the study.

**Table 1:** Inclusion and exclusion criteria Meta-analysis is a quantitative, formal, epidemiological study design used to systematically assess the results of previous research to derive conclusions about that body of research; randomized control trials (RCTs) are prospective studies that measure the effectiveness of a new intervention or treatment.

Inclusion Criteria	Exclusion Criteria
2018 to 2023	Grey literature
Middle-aged: 45 years and above	Unpublished literature
Full free text	Animal studies
Human	Duplicates found and removed
Male and female	Studies excluded are Phase 2 Trials and below
English Clinical Trial Meta-analysis	
Randomized controlled trials (RCTs)	
Review	
Studies with patients using at least one of both drugs	
Studies included are Phase 3 Trials and above	

Results

**Figure 2:**
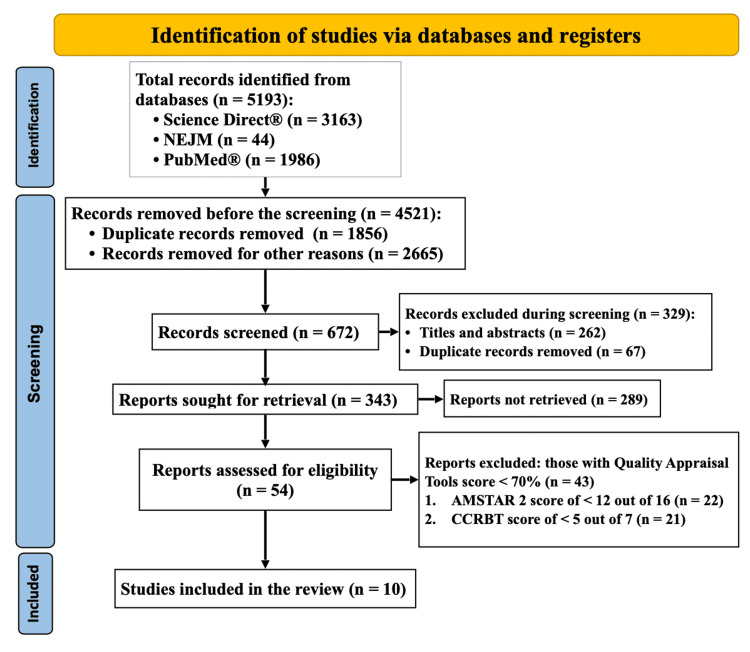
Displays the PRISMA flowchart of the literature and the study's search methodology Number (n); Preferred Reporting Items for Systematic Reviews and Meta-Analyses (PRISMA); Cochrane Collaboration Risk of Bias Tool (CCRBT); ScienceDirect is the world's leading source for scientific, technical, and medical research; idiopathic pulmonary fibrosis (IPF); the New England Journal of Medicine (NEJM); PubMed comprises more than 36 million citations for biomedical literature, life science journals, and online books.

Using the standard keywords, 54 pertinent publications were located in ScienceDirect, the New England Journal of Medicine, and PubMed. The selection process involved finding and removing duplicates and all the grey literature on the subject and literature based on animal studies. A comprehensive set of 5193 studies was identified from different databases of ScienceDirect (3163), PubMed (1986), and the NEJM (44). After eradicating 1856 duplicate records and 2665 records for various other reasons, 672 records were left. After titles and abstracts were screened, we were left with 54 relevant studies, of which 43 were excluded as they were irrelevant to the study. After a full-text screening, only ten papers that matched the criteria for this study topic and were relevant were found. PRISMA flowchart of the literature and the study's search methodology [11] is shown in Figure 2.

Study Characteristics

To understand their effectiveness, an evaluation of 10 previously circulated papers of patients detected with IPF and being treated with nintedanib and pirfenidone was done. A total of 10 studies were included in this collection. In all the studies, the comparison was made between patients suffering from IPF and on treatment with either nintedanib or pirfenidone or both versus patients suffering from IPF on a placebo. The studies included patients of different ages, sexes, and ethnicities worldwide, and the observations were noted. It was observed that, across the ten studies, patients exhibited a reduced decline in lung function (FVC) when treated with either nintedanib, pirfenidone, or both, compared to those administered a placebo. However, the adverse effects on patients being treated with nintedanib or pirfenidone or both were much higher than on patients being given a placebo. A detailed summary of the studies included in the study is shown in Table 2.

**Table 2:** Summary of the included papers Composite physiologic index (CPI); the symbol for milligram, an SI unit of mass equal to 10−3 grams (mg); an SI unit for volume, 1 mL is equal to one-thousandth of an L (1/1000 L) (mL); diffusing capacity of the lung for carbon monoxide (DLCO); forced vital capacity (FVC); oxygen saturation by pulse oximetry (Sp02); gender-age-physiology (GAP); idiopathic pulmonary fibrosis (IPF); St. George's Respiratory Questionnaire (SGRQ); health-related quality of life (HRQOL); patient-reported outcome (PRO); standard error (SE); Patient Experiences and Satisfaction with Medications (PESaM); treatment-emergent adverse events (TEAEs); six-minute walking distance (6MWD).

Authors (year)	Design	Sample size (male and female)	Intervention	Follow-up period	Outcomes measured
Kreuter et al., 2020 [12]	Two INPULSIS® studies of 52 weeks, randomized, double-blind, placebo-controlled, parallel-group Phase III trials	Nintedanib = 638; placebo = 423	Nintedanib 150 mg twice daily	52 weeks	Nintedanib slowed deterioration among patients with advanced disease at the baseline (defined as GAP II/II, FVC % ≤ 80%, DLCO % ≤ 40%, CPI > 45, or SGRQ > 40) HRQOL and symptoms as assessed by several PROs.
Richeldi et al., 2020 [13]	Clinical trials conducted in patients in INPULSIS® and INSTAGE trials	Nintedanib INPULSIS = 638; INSTAGE trials = 136; placebo INPULSIS® = 423	Nintedanib 150 mg twice daily	24 weeks	Rate of FVC decline with nintedanib INPULSIS® = -52.3 mL/24 weeks and in INSTAGE = -66.7 mL/24 weeks; rate of FVC decline in placebo INPULSIS® = -102.8 mL/24 weeks; acute exacerbations in patients with nintedanib INPULSIS® = 0.6% and in INSTAGE = 3.7%; acute exacerbations in placebo INPULSIS® = 2.1%; deaths in patients using nintedanib INPULSIS® = 2.0% and INSTAGE = 11%; deaths in patients in placebo INPULSIS® = 1.9%; adverse events (diarrhea) with nintedanib INPULSIS® = 52.5% and in INSTAGE = 48.5%; adverse events (diarrhea) in placebo INPULSIS® = 16.1%
Flaherty, 2018 [14]	Clinical trials conducted in patients in INPULSIS® trials	Nintedanib = 519; placebo = 345	Nintedanib 150 mg twice daily	52 weeks	191 patients (36.8%) treated with nintedanib showed improvement or no decline in FVC of whom 181 had an improvement; 62 patients (18.0%) in the placebo group had an improvement or no decline in FVC of whom 55 had an improvement.
Song et al., 2020 [15]	Randomized Controlled Trial INPULSIS® - ON in Asian patients of different ages and sex	INPULSIS®- ON nintedanib = 215: continued nintedanib in INPULSIS® ON = 121; initiated nintedanib in INPULSIS® - ON having received placebo in an INPULSIS® trial = 94	Nintedanib 150 mg twice daily	52 weeks, follow up after 192 weeks	INPULSIS® trials' annual rate (SE) decline in FVC on using nintedanib = -124 mL (20)/year; placebo = -218 mL (24)/year; INPULSIS® ‐ ON annual rate (SE) decline in FVC on nintedanib = -127 (11) mL/year in Asian patients.
Moor et al., 2020 [16]	Randomized Controlled Trial prospectively completed with PESaM	Nintedanib = 39; pirfenidone = 51	Nintedanib and pirfenidone	12 weeks nintedanib and 24 weeks pirfenidone	The effectiveness of antifibrotic treatment of both nintedanib and pirfenidone was similar at the end of six months. Adverse effects experienced by patients on nintedanib were mainly gastrointestinal like diarrhea, abdominal pain, and tiredness, and by patients on pirfenidone were fatigue, skin-related, and low appetite.
Brown et al., 2019 [17]	Clinical trial INPULSIS®	Nintedanib = 638; placebo = 423	Nintedanib 150 mg twice daily	52 weeks	Nintedanib showed a significant decrease in FVC% versus placebo. There was no difference in DLCO%, CPI, or SpO2 between nintedanib and placebo.
Nathan et al., 2019 [18]	Clinical trial ASCEND and CAPACITY	Pirfenidone = 90; placebo = 80 (127 patients in ASCEND and 43 patients in CAPACITY)	Pirfenidone 2,403 mg/day	52 weeks	It was observed that the patients treated with pirfenidone showed more than a 10% decline in FVC than the patients treated with a placebo after following up after 52 weeks. Even with TEAEs, the discontinuation of the treatment was low.
Nathan et al., 2019 [19]	Clinical trial (post hoc exploratory analysis of disease progression)	Pirfenidone = 623; placebo = 624	Pirfenidone 2,403 mg/day	52 weeks, follow up after 194 weeks	The disease progression events assessed in the study were a % decline in FVC and six-minute walking distance (6MWD). It was observed that the patients who received pirfenidone had more than one progression event. Deaths reported were fewer compared to the placebo group.
Glassberg, 2019 [20]	Clinical trial (post hoc analysis of pirfenidone on dyspnea severity)	Pirfenidone = 617; placebo = 617	Pirfenidone 2,403 mg/day	52 weeks	It was observed that pirfenidone reduced the episodes of dyspnea in patients who showed FVC < 80% or GAP stage II/III. The benefit of dyspnea reduction was not observed much in patients with preserved FVC and GAP stage I.
Maher et al., 2019 [21]	Randomized Controlled Trial (CAPACITY and RECAP studies)	Pirfenidone = 236; placebo = 249	Pirfenidone 2,403 mg/day	220 weeks	Pirfenidone showed efficacy in reducing the decline in lung function after its initiation; also it was not able to restore the already damaged function of the lung. Thus, the timing for initiating the treatment is beneficial to the patient with IPF irrespective of the stage of IPF.

Discussion

Nintedanib: Mechanism of Action

To treat patients with IPF, the United States of America has approved the use of the tyrosine kinase receptor inhibitor, nintedanib. By October 2014, both the US Food and Drug Administration (FDA) and the European Medicines Agency (EMA) approved the drug nintedanib for the treatment of IPF [22]. It works by inhibiting the capacity of growth factor receptors, such as vascular endothelial growth factor (VEGF), platelet-derived growth factor (PDGF), and fibroblast growth factor (FGF), to kinase [23,24]. It stops fibroblasts and myofibroblasts from activating, which would otherwise lead to an excess of extracellular matrix proteins and fibrotic scar tissue [5,6]. Nintedanib decreased FVC irrespective of dose adjustments made in patients due to the development of severe adverse effects and had similar outcomes in both males and females, irrespective of age. One hundred ninety-one patients (36.8%) treated with nintedanib showed improvement or no decline in FVC, of whom 181 had an improvement. Sixty-two patients (18.0%) in the placebo group had an improvement or no decline in FVC, of whom 55 had an improvement [14]. Nintedanib showed a significant decrease in FVC% versus placebo. There was no difference in the diffusing capacity of the lungs for carbon monoxide (DLCO)%, composite physiologic index (CPI), or oxygen saturation by pulse oximetry (SpO2) between nintedanib and placebo [17]. Nintedanib effectively decreased the progression of patients' severe illness at the baseline described as Gender-Age-Physiology (GAP) II/III, CPI ≤ 45, DLCO ≤ 40%, FVC ≤ 80%, or St. George's Respiratory Questionnaire (SGRQ) > 40 [12]. It positively affected their health-related quality of life (HRQOL) and symptoms as evaluated by several patient-reported outcomes (PROs) in two INPULSIS® Phase III studies [12,13]. INPULSIS® study design includes randomized, double-blind, placebo-controlled, parallel-group trials in 24 countries across the Americas, Europe, Asia, and Australia. Nintedanib is a drug that has been shown to improve the physiological functioning of the lung and to arrest the progression of lung fibrosis in IPF patients. Also, in the patients suffering from advanced IPF, treatment with nintedanib resulted in less deterioration in HRQOL [12]. 

Using Nintedanib

The dosage of nintedanib used to treat patients with IPF was 150 mg twice daily. The effectiveness of the medication in halting lung fibrosis advancement or further decline in FVC in patients with IPF was studied in six studies. In light of the previously given Table 2 and due to worries about the safety, efficacy, and associated side effects of antifibrotic medications in these patients, as well as issues with patients who are elderly and have accompanying comorbidities, doctors are still hesitant to treat patients with advanced IPF, even though nintedanib treatment results in a physiological improvement of lung function [14].

Additionally, Song et al. ascertained during INPULSIS® trials that the annual rate standard error (SE) decline in FVC with nintedanib = -124 mL (20/yr), Placebo = -218 mL (24/yr), nintedanib-induced INPULSIS®-ON annual rate (SE) decline in FVC = -127 mL (11 /yr) [15,16]. In both trials, as shown in Table 2, the annual rate of decline in FVC after treatment with nintedanib was much greater than placebo.

Pirfenidone: Mechanism of Action

Uncertainty surrounds the precise mechanism of action of pirfenidone. However, it is believed to have antioxidant, anti-inflammatory, and antifibrotic properties [18,19]. It is used as a drug to treat IPF because it is thought to inhibit fibroblast growth, their transformation into myofibroblasts, and the generation of collagen, demonstrating some antifibrotic efficacy [25,26]. It was observed that the patients treated with pirfenidone showed more than a 10% decline in FVC than the patients treated with a placebo after following up after 52 weeks. Even with treatment-emergent adverse events (TEAEs), the discontinuation of the treatment was low [18].

The disease progression events assessed in the study were a % decline in FVC and six-minute walking distance (6MWD). It was observed that the patients who received pirfenidone had more than one progression event. Deaths reported were fewer compared to the placebo group. It was observed that pirfenidone reduced the episodes of dyspnea in patients who showed FVC < 80% or GAP stage II/III. The benefit of dyspnea reduction was not observed much in patients with preserved FVC and GAP stage I [20].

Pirfenidone showed efficacy in reducing the decline in lung function after its initiation, but it was not able to restore the already damaged function of the lung. Thus, the timing for initiating the treatment is beneficial to the patient with IPF, irrespective of the stage of IPF [21].

Using Pirfenidone

Pirfenidone exhibited positive results in treating IPF patients by hindering the progress of fibrosis in patients in four separate studies. According to the study by Nathan et al., in a clinical trial with pirfenidone versus placebo, the patients treated with pirfenidone (dose of pirfenidone = 2,403 mg/d) exhibited a greater reduction in FVC than the patients treated with placebo after following up after 52 weeks [19].

Even with treatment-emergent adverse events (TEAEs), the rate of treatment discontinuation was not excessive [18]. In addition to lowering FVC %, pirfenidone was also discovered to minimize progression events, dyspnea (in GAP II/IlI stage), and six-minute walking distance (6MWD), according to a study by Nathan et al. [19] and Glassberg [20] with a follow-up period of 12 months.

The findings of a Randomized Controlled Trial CAPACITY and RECAP done by Maher et al. [21], who completed the clinical studies assessing pirfenidone in IPF, showed that the timing of drug initiation, independent of the stage of IPF, is beneficial to the patient. CAPACITY is a multinational phase 3 trial conducting clinical studies assessing the research of efficacy and safety outcomes of pirfenidone in IPF, and RECAP is an open-label extension study evaluating the long-term safety of pirfenidone in patients with idiopathic pulmonary fibrosis. Pirfenidone demonstrated success in delaying the loss of lung function after it was started, but it was unable to heal the previously damaged lung [21].

Relative Efficacy of Nintedanib and Pirfenidone Therapy and Patients' Reactions

Nintedanib and pirfenidone both demonstrated equivalent antifibrotic therapeutic efficacy after six months, according to research by Moor et al. [16]. Patients taking pirfenidone experienced fatigue, lethargy, and skin-related adverse effects, but those on nintedanib predominantly had gastrointestinal side effects, such as diarrhea. Because of such adverse side effects in some IPF patients, the treatment's suspension had to be done [27].

In the first-ever real-world comparative observational study, Cerri et al. [28], investigated the effects of the two drugs in IPF patients. They discovered that over a 24-month period, nintedanib and pirfenidone delayed the decline in FVC and DLCO in comparison to nontreated individuals [28]. However, in different countries, there are varied views when it comes to the treatment of IPF with the presently available drugs, mainly because of a lack of communication between the patients and physicians [29].

Limitations

Lack of clarity regarding the impact of the drug nintedanib on patients of IPF when used alone or when it was added to pirfenidone is indeed a limitation of this study. Further study is required to understand the safety and tolerability of nintedanib when used alone and when it is added to pirfenidone in patients with IPF.

The real-world patients are usually different from those in clinical trials as the real-world patients have a higher rate of comorbidities like diabetes, ischemic heart disease, and hypertension. The access to lung function results before and after treatment is less in real-world patients than in clinical trial patients. Therefore, missing data reduces the clarity in deducing the effects of these drugs on patients of IPF.

As most of the patients suffering from IPF are older and have more comorbidities as compared to those in clinical trials, the proper investigation and study of the safety and tolerability of the drugs nintedanib and pirfenidone in a real-world setting requires a larger sample spread over many countries mainly because the present survival rate in IPF patients is very low.

## Conclusions

Nintedanib and pirfenidone have been used to remedy IPF effectively worldwide. Both drugs have demonstrated the capacity to postpone the beginning stages of FVC decrease and lung function decline. Despite the fact that there is no known etiology for IPF, this research has demonstrated that there are numerous factors that need to be taken into account when treating the condition. These factors include the patient's comorbidities, age, and capacity to endure the treatment's negative effects. Clinical trials done worldwide have shown that the usage of both medications improved the therapy of IPF overall irrespective of age, gender, and ethnicity. With the exception of the negative effects that the patients who took each drug separately had experienced, there was little difference between the efficacy of nintedanib and pirfenidone. Both drugs were safe and well tolerated for the treatment of IPF. However, there is no treatment yet available to treat the already damaged lung which worsens with the passage of time. Future research should focus on early detection of the disease and timely initiation of the administration of the drugs, reversal of the fibrosis in the lungs, and lowering patient mortality from IPF.
